# Opening Address at the Southern Medical College, Octocer 1st

**Published:** 1895-11

**Authors:** Henry F. Harris

**Affiliations:** Atlanta, Ga.; Professor of Chemistry


ATLANTA

### Medical and Surgical Journal.

VOL. XII. NOVEMBER, 1895. No. 9.

LUTHER B. GRANDY, M.D., MANAGING EDITOR.

M. B. HUTCHINS, M.D., BUSINESS MANAGER.

COLLABORATORS:

H. V. M. MILLER, M.D., LL.D., VIRGIL O. HARDON, M.D., FLOYD W. McRAE, M.D., DUNBAR ROY, M.D., AND H. R. SLACK, M.D., PH.M.

### *ORIGINAL COMMUNICATIONS.*

### OPENING ADDRESS AT THE SOUTHERN MEDICAL COLLEGE, OCTOBER 1ST.

BY HENRY F. HARRIS, M.D.,

Professor of Chemistry.

I will speak to you to-day of the lives and deeds of some of those whose labors have made our profession what it is. We cannot too often be reminded of these mighty ones who have gone before us, nor is anything of more intense interest to every physician who has the good of his profession really at heart. Of almost equal interest to all should their lives be, for it should never be forgotten, as it too often has been, that they worked and often sacrificed everything, even life itself, for the good of their fellow-men. Their glorious deeds belong exclusively to none, but are the common heritage of mankind. In marked contrast to the great good which they have accomplished is the utter want of appreciation of it which is shown by the world. By what unaccountable impulse do we vie with each other in doing honor to those scourgers of mankind, who, from time to time, to satisfy their lust for power, have deluged the world with blood? By what strange perversion of all right thinking are their names placed first on the brightest pages of the histories of all nations, while are left to oblivion those who, though simple and unassuming, have fought in solitude the great battles against pain and death with equal courage—nay, often with even greater, for there were no onlookers to encourage or to applaud.

However, to us their humble followers and admirers every word, every action, every thought of theirs is of the deepest significance; even now, in spirit we accompany them on their daily rounds, we bend with them over the couch of suffering and of death, and read in their sympathizing eyes that love and unutterable pity which have made so many of them martyrs to the welfare of the human race; we weep with them in their sorrows, we rejoice with them in their good fortunes, and in their God-like deeds we render them that homage which no emperor ever commanded—for they are the masters of our hearts. Let us then, to-day, devote the short time which has been accorded to us to recalling the achievements and scattering immortelles o'er the graves of some of those to whom we owe so much, and be assured that we cannot come away without being bettered.

From the earliest times the healing art has been more or less practiced by all nations, often in connection with religion—the office of priest and physician being generally combined. So it continued until the fourth century B. C., when there arose in Greece—the great center of thought and civilization of antiquity—a school of medicine, the influence of which is even felt to the present day. The founder of this school, and chief among the physicians of ancient times, was Hippocrates of Cos. Born of a family of priest-physicians, the asclepiadæ, and believed to be a direct descendant of Æsculapius, the Greek god of medicine, he was early initiated in all the mysteries of the art as then known, and at the same time was thoroughly inculcated with the religious superstitions with which medicine was at that time so inextricably united. Fortunately he lived in the age of the greatest intellectual development of his country; he had as his teacher Democritus, the originator of the doctrine of atoms, and there can be no doubt that he was greatly influenced by the teachings and writings of Sophocles and Thucydides, of Socrates and Plato, who were his contemporaries, probably his friends.

In one of his vast mental power, subject to such influences, it is not surprising that the bonds of superstition and prejudice were burst asunder, and that he it was who, first in the history of the world, began to build up the science of medicine in the only possible way—namely, by the close and accurate observation of the phenomena of health and disease. So accurate was he in the delineation of the symptoms of disease, that many of his descriptions remain to the present day models of exact clinical observation. As an example of this may be mentioned his discovery of the succussion sounds which may be produced in the chest in some diseases, and which foreshadowed—nay, even suggested—the brilliant discoveries made in this direction in modern times. Nor was he wanting in the graphic power necessary to give interest to his writings, as is illustrated by his masterly description of the distorted facial expressions of the dying, and known since his day as the "facies Hippocratica." He it was who likewise first taught the rational treatment of disease, and insisted that the physician's office was only to assist nature. He rebelled against charms, amulets and prayer as remedial agents, and held that however much the gods were responsible for disease, that they were subject to natural laws and should be treated accordingly. Notwithstanding his great practical sense and his aversion to theory, his knowledge of the fundamental branches of medicine was too meager to prevent his falling into the error of giving support to hypotheses without foundation.

The one which exercised the greatest influence on medical thought, and, in truth, almost the only explanation for disease offered during several centuries following, was his humoral pathology; according to this dogma the body contains four humors—blood, phlegm, yellow bile and black bile, the increase or the decrease of one or the other, or the irregular distribution of either producing the phenomena of disease. But these erroneous theories were comparatively few, and his positive contributions were of such immense value, that he holds undisputed to the present day the proud title of the Father of Medicine. He was a man of the greatest moral worth, and died at an old age universally venerated by his countrymen.

After the death of Hippocrates, medicine in Greece, along with other intellectual pursuits, suffered a gradual decline. The distracted condition of the country caused the few men of scientific ability which it produced, to seek safety and protection in Egypt. Here, under the wise and beneficent rule of Ptolemy Soter and his immediate successors, Philadelphus and Euergetes, lived and flourished Herophilus and Erasistratus, who gave us exact anatomical knowledge of the human body, owing to the enlightened policies of these monarchs who first permitted dissection. Herophilus, especially, discovered many new anatomical facts, and of the Greek physicians was esteemed only second to Hippocrates.

Following these physicians, no one of great note appeared until we arrive at the period when the greatness of Rome attracted to itself what remained of Greek culture. Although several Greek physicians of eminence settled in Rome, the only one who may be said to have left any permanent impression on medical thought was Asclepiades, the friend of Cicero. The principal interest which he has for us is that he opposed the humoral pathology of Hippocrates by a system, equally without facts to substantiate it at that time, which gave as an explanation of disease, that the atoms of which the body is composed are altered in size, number, arrangement or movement. In this we see dimly foreshadowed our modern demonstrations of the chemical changes which occur in disease. The greatest of his disciples was Soranus of Ephesus, who lived in the second century of the Christian era. He devoted himself almost entirely to the study of diseases of women, and his work on this subject, which is the only complete one of its kind which has come down to us from antiquity, shows him to have been an enthusiast and to have possessed great practical knowledge of his subject. He was acquainted with the use of the speculum, which has been reinvented in our own day.

Contemporaneously with Soranus lived Galen, who, among the ancients, stands second only to Hippocrates, and whose teachings almost completely dominated the medical mind up to two or three hundred years ago; even now it can scarcely be said that we are entirely emancipated from his influence. Galen was thoroughly versed in all of the philosophy and science of his time, but more particularly devoted himself to medical studies. A disciple of Hippocrates, he enlarged and extended his theoretical dogmas with such marvelous ingenuity, that its effect in the end was to even overreach itself.

He invented with the greatest readiness names for the various phenomena which came under his observation, and was never at a loss for explanations of the most difficult problems. He was, however, an enthusiastic anatomist, and his writings show that he possessed great knowledge of medicine. Strange to say, it was not for these more solid qualities that he has been mostly esteemed, but rather for his speculations and fine-spun theories which were admirably suited to the mental attitude of succeeding ages.

In the centuries following the death of Galen medicine participated in the general decay of knowledge which characterized these times, and, although some writers of note were produced, the works of none were of sufficient importance to produce a lasting impression. Thus matters stood, when in the early infancy of the art of printing—the invention of which was the signal for the universal intellectual awakening of the world—at the time when the mighty genius of Copernicus was roaming alone and unguided over the boundless oceans of space and marking out the pathways of the stars, when soon after the unequaled courage and genius of Columbus had presented his royal masters with a new world and furnished the first positive proofs of the shape of our globe—at this time, as we should have expected, medicine aroused itself from its long lethargy, and, never more to recede, with invincible strength will keep its triumphant way until pain and suffering and disease shall have ceased to be. From the lofty heights of the mountains of Switzerland modern medicine took its birth in the person of a strange and eccentric genius—Theophrastus von Hohenheim, by name—but commonly known as Paracelsus, an appellation which he assumed in later life. When still young he was instructed in the rudiments of medicine by his father, himself a physician, which he acquired with great rapidity, but early showed a disposition to doubt the truth of what had been taught him and to observe for himself. With the exception of Hippocrates he exhibited always a great contempt for the earlier physicians ; he was accustomed to assert that his shoe-buckles were more learned than Galen, and even began his university course in medicine by burning all his books. After graduating he largely pursued chemical studies and was the first to approach a correct idea of the relation which chemistry bore to vital processes. He was the first to prepare laudanum, and after much experimenting, succeeded in making several new chemical compounds. Merciless in the condemnation of the dogmas of others, no one ever originated more wild and unfounded theories than he.

After all, his chief merit—and this is very great—lies in his fearless protests against the vagaries of his time, and in partially substituting the inductive for the deductive method of his predecessors. With some justice he has been called the Luther of medicine.

The next great forward step was the discovery of the circulation of the blood by Harvey, which was published in 1628, but in his lectures his views had been put forth a dozen years earlier.

An ardent student of anatomy and a profound thinker, his immortal discovery was conceived while still a young man, and to his everlasting credit be it said—which could be said of none before him—that he spent more than half a lifetime in collecting every possible proof and in the most painstaking and tedious experiments to prove the truth of this most important and fundamental of all physiological phenomena. Harvey was the first true representative of modern medicine, and in point both of ability and the methods he employed compared favorably with any of his successors.

He died at an old age without children, and left the bulk of his property for the endowment ot an annual oration; he instructs the orator "to exhort the fellows of the college to search out and study the secrets of nature by way of experiment, and also for the honor of the profession to continue mutual love and affection among themselves." No better and few greater men have ever lived.

In the meantime knowledge of anatomy had gradually but steadily grown—each generation adding to what had been learned in the last. As yet, however, only gross anatomy—that which could be seen with the unaided eye—was known, and a knowledge of those finer but none the less interesting details was still to be attained.

About the middle of the seventeenth century the increasing use of the microscope, at that time very imperfect, caused a grinder of lenses—Anthony von Leeuwenhoek—and a resident of his native city of Delft, in Holland—to use this instrument for examining blood, tissues of the bodies of animals, milk and many other natural objects with the most astonishing results; he found that the blood was largely made up of red disks, which he correctly described; he discovered the fat globules in milk, the epidermis of the skin, the fibrous character of the crystalline lens, the tubules of the teeth, and many other things too numerous to mention. He was the first to take stand against the prevalent opinion of spontaneous generation, and very largely proved the truth of his views. Against the superstition and false theories of his time he waged unceasing warfare, so that, while not a physician, perhaps no one ever lived whose influence was so important in cleansing medicine, with kindred scientific subjects, of their theoretical rubbish, and substituting in its place a solid foundation of unalterable truths. While it is true that the pre-eminence of Leeuwenhoek in his special line of investigation was in a measure due to circumstances, there is no question of the purely scientific spirit which actuated him, and his clear and convincing demonstrations, with the absolute justness of his conclusions respecting them, will forever remain models of the scientific method.

He died at an extremely old age, universally respected, and leaving behind him a reputation which will die only with our civilization. With justice he is considered the father of scientific microscopy.

Another name inseparably connected with that of Leeuwenhoek by the common direction of their investigations is that of his contemporary Malpighi. Born near Bologna, he studied medicine at the university of that city and was graduated at the age of twenty-five. He soon after began the microscopical study of the tissues and internal organs of man and the higher animals with the most brilliant results. He accurately described a layer of the skin, the convoluted masses of blood-vessels in the cortex of the kidney, and adenoid masses in the spleen—all of which received his name. Four years after the death of Harvey he demonstrated in the lungs the capillary vessels through which the blood flows from the arteries to the veins—thus completing the work of this great investigator who had only inferred the existence of these minute vessels; he shortly after gave a description of the minute anatomy of the lung, which first made possible an understanding of the respiratory act. Although he studied somewhat the minute anatomy of plants, unlike Leeuwenhoek he devoted himself almost entirely to the investigation of animal tissues, and for this reason is generally considered the originator of microscopical anatomy. He was a professor in the university from which he graduated for twenty-five years. As an example of the fierce and stubborn resistance with which ignorance opposed every advancement in medicine in its earlier days may be cited the fact that some of the university colleagues of Malpighi regarded his microscopical studies as entirely opposed to the best interests of medicine, and so extreme did this antagonism become that it occasioned a duel between a brother of Malpighi and a near relative of one of his opponents. It is some satisfaction, even at this day, to know that Malpighi's champion, while escaping himself entirely unhurt, killed his adversary *secundum artem.* Thus did young medicine vigorously assert itself, but it was a sad and unnatural beginning for a science whose object before everything else is to preserve life.

A few years older than either of the two preceding was Thomas Sydenham. Like Harvey he was an Englishman, but unlike him was quite as staunch an adherent of Cromwell as the other was devoted to the fortunes of the unfortunate Charles. His medical studies were even for a time interrupted to permit his serving in the army of Parliament. At the age of twenty-four he graduated as bachelor of medicine. He was a man of the greatest practical sense, was hampered by no theories, and with but scant exceptions drew all his lessons from nature. He admired greatly Hippocrates, but had little respect for any of the others who had preceded him. He cared nothing for anatomy, and knew nothing of abnormal anatomy—which had not been studied in his day. He insisted that all forms of ill-health had their special types—a fact which before his time had been but vaguely understood—and it was his constant aim to so classify them that they might be easily recognized.

In his treatment of disease he depended greatly on the powers of nature—sometimes using no drugs at all—and advocated always simple methods. He did for practical medicine very nearly what had been done for both gross and minute anatomy by former and contemporary anatomists. Sydenham's accurate observations on the natural history of disease were epoch-making, and while they gained for him at once the friendship and high esteem of such great men as Locke and Boyle, they were too far in advance of his time to be at once generally appreciated. He was essentially a man of thought, which, coupled with his absolute sincerity, has caused him to appear greater and greater with the onward roll of time, until now, with no superiors and few equals, he stands a mighty beacon tower, the light of which has since guided the timid and faltering footsteps of our science in its never ending search for truth. It is no false compliment to call him the English Hippocrates.

The seventeenth century, which, within a decade, had brought forth the three illustrious men of whom I have just spoken, toward its close gave birth to a fourth who rivalled, if he did not eclipse, either of the others. It is of the renowned Morgagni that I speak, the founder of the science of morbid anatomy. Like Malpighi, an Italian, and also a graduate of the University of Bologna, he seems to have made at once that favorable impression on the faculty of that institution which he never failed to produce in later life. He was prosecutor for Valsalva, whom he assisted in preparing his celebrated work on the anatomy and diseases of the ear, and a year or so later succeeded him as demonstrator of anatomy to the University. In his thirty-first year he accepted a professorship in the University of Padua, and three years later was appointed to the chair of anatomy at the same place. Here he taught for nearly sixty years, and during this time began that series of masterly investigations which resulted in the creation of a new science, the importance of which to medicine it is impossible to overestimate. Before his time anatomy, as we have seen, had been assiduously cultivated, but no one had as yet taken the trouble to study the changes in the organs and tissues which occurred in disease ; to this great work Morgagni devoted himself with a patience, enthusiasm and brilliancy which has been very rarely equaled—certainly never excelled. Like Sydenham—of whom he was in many particulars the counterpart—he had little inclination toward theorizing and depended almost entirely on facts to gain knowledge. In his eightieth year he published his great work on abnormal anatomy, thus embracing the minute and accurate observations of a lifetime, which alone was quite comprehensive enough to place the science on a secure foundation. Morgagni was a man of the rarest social gifts and personal magnetism ; his popularity with all classes was without a precedent, and has remained without example to the present day. The Venetian senate from time to time raised his stipend, until he was paid more than any professor in this University had ever received, but he was so highly esteemed by his colleagues that it is said never to have occasioned the least jealousy. He was honored by successive popes, and on two occasions when his city was occupied by hostile armies his house was treated with the same marked distinction which Alexander the Great showed to the house of Pindar. At the age of eighty-nine he died—ten years after the publication of his immortal work, and with him perished the last of those great men whose efforts had successively molded into definite shape the fundamental branches of the science of medicine. * * * * * *

Since the laying of its foundations many masterful workmen have labored on this magnificent temple of Æsculapius—workmen who have reared a superstructure, the grandeur of which is in every way worthy of its beginning. Let us briefly take up the records and see what these children of Paracelsus and Sydenham, of Morgagni and Harvey, of Leeuwenhoek and Malpighi, have accomplished to make them worthy of such illustrious ancestors.

About the year 1769, a medical student, Edward Jenner by name, living in the small town of Sodbury, England, learned that it was commonly believed in the surrounding rural districts that a disease of the cows, called cow-pox, could be contracted by man, and that those who had suffered in this way were incapable after of contracting that fearful scourge, the small-pox. Being a youth of thoughtful habits, this popular belief, although it seemed highly improbable, frequently recurred to him during his student days in London, where he was a favorite pupil of John Hunter, the celebrated physiologist and comparative anatomist. After completing his medical studies, he returned to his native village and again began his inquiries on the above subject. Many were the difficulties, but with a courage and determination born of high purpose, combined with great natural ability, he overcame all obstacles and finally satisfied himself of the truth of this curious and most important fact. Almost exactly one hundred years ago, he demonstrated the truth of his great discovery by vaccinating a boy and later proving that he was immune to the terrible small-pox. Slowly at first, but later with great rapidity, this most important of all human discoveries spread over the civilized world, opposing an invisible but impenetrable shield to the attacks of the most loathsome and fatal of all the enemies of mankind.

Wernher, in his book on vaccination, says: "Before the introduction of vaccination, small-pox had become a permanent disease which never entirely ceased in any one year, and every three or five years became a great epidemic. Very few men escape small-pox until old; almost every one sickened at least once in his life of this horrible and murderous disease. Countless mortals were maimed by loss of sight; of new-born children one-third died before their first year, one-half before their fifth year."

Dr. Lettsom calculated that, in Europe alone, two hundred and ten thousand beings were, from this cause, deprived of life each year. The mortality was much greater in barbarous and semi-civilized countries. Living under the beneficent protection of vaccination, we can form no conception of the terror which this dread disease at one time inspired, and we accept our immunity in the same matter of-fact-way that we do water or the air—without once inquiring from whence it came. Jenner's merits were, in a measure at least, recognized before his death, and, strange to say, much more in foreign countries than in his own. During the troubled times of the latter part of the last century and the beginning of the present, no name on the European continent inspired so much respect as that of Jenner. His friends, before leaving for foreign countries, often procured passports signed by him, and to the everlasting credit of human nature, they were invariably honored by all parties as none others would have been. In cases where the combined strength of Europe would have been powerless, and after all other measures had utterly failed, the simple requests of Jenner to the Great Napoleon were invariably effectual. The greater part of what remained of his active years were employed in perfecting his work and defending it against ignorance and prejudice—a labor which he conscientiously performed from a high sense of duty rather than from any inclination toward it. And after all was finished, with constantly increasing reputation both at home and abroad, the evening of his life passed quietly away in congenial intellectual pursuits. He died January 26th, 1823, at the age of seventy-four, universally honored and blessed by his fellow-men. He had achieved the highest possibility of human destiny, for he was "both old enough and good enough to die."

Could there be possibly a more effectual answer than this man's life to any one who might be disposed to deny the value of painstaking observation?

Bear with me a little longer and let us see what can be accomplished by combining the methods of Sydenham and Morgagni. On the 17th day of February, 1781, there was born on the precipitous shores of the bay of Biscay, in lower Brittany, in France, a child whose name was destined to be often mentioned in succeeding ages. From birth very small and of mean appearance, he gave but little promise of surviving childhood—much less was there any indication of the extraordinary fate which the future had in store for him. But even then he seemed, as he always did, to defy circumstances, and lived through the perils which surround childhood despite the sickly and feeble frame which nature had given him. Roaming at will over the desolate moorlands and climbing the almost mountainous crags of his native province, his strength slowly increased with years, so that, although he was always diminutive in size, in adult life was capable of remarkable endurance. But if his body obstinately refused to grow, it was more than compensated for by the giant strides of his mind—for he early showed great strength and precocity of intellect. At about the age of seventeen he began the study of medicine under an uncle, and two years later went to Paris to complete his medical education. Here he devoted himself to everything connected with medicine with an ability and perseverance which almost passes belief. During the first three years of his student life he recorded the histories of nearly four hundred cases of diseases with the utmost minuteness. During this time he made several minor anatomical discoveries, wrote for publication descriptions of interesting cases of disease which came under his observation, was the author of an essay on peritonitis which was far in advance of his time, and actually began a highly successful course of lectures on morbid anatomy before obtaining his degree. After graduating he continued to work just as he had before, but it was not until twelve years later that he made the great discovery which at once placed his name on equality with the greatest of those who had preceded him; this consisted in finding out the fact that the heart and lungs produced certain sounds while performing their functions, which in health were always practically the same, and that these sounds could be heard when the ear was applied to the chest; he noted that they underwent certain modifications in disease, and that even sounds altogether new might be produced. To the task of finding out in just what conditions of the heart and lungs these new or modified sounds were produced he now addressed himself. Making the most painstaking examinations of patients during life, he noted how the symptoms and sounds corresponded with the diseased states found in his post-mortems; with feverish energy he toiled unceasingly, as if conscious that what he had to do must be done quickly—that the race of life was almost run. If he had accomplished nothing more, either his observations on the symptoms of disease or his investigations into morbid anatomy would have been quite enough to make him famous. But he did far more: under the creative power of his genius physical diagnosis sprang up as if by magic, and, in an incredibly short space of time, we were presented with the rare spectacle of a science in almost complete perfection given to the world by its originator. But his health, always precarious, seemed to decline from the very hour he began the crowning work of his life, so that we find him instantly quitting Paris, after its completion, with the hope of restoring his shattered constitution amid the scenes of his youth; here he rallied for a time, but it soon became evident that it was too late. Fanned by the breezes which had given his young life strength, surrounded by the objects which were to him the dearest on earth, in the very heyday of his existence, when by the voice of universal fame crowned king, perished the immortal Laennec, of the very disease about which he had taught us more than all others. His poor, sickly body has gone back to the dust from whence it came, but his spirit lives. So enormous were his practical contributions to medicine that we never examine a patient without making use of some of his methods or something he taught us; before his time all was guesswork, since, all exactness. He was the very incarnation of the scientific spirit of all ages, and in his short life taught us far more than any other one man who ever lived. His charity and goodness were as boundless as his genius; the supplications of poverty and want were never addressed to him in vain; the rich and powerful he often refused to visit, the poor and lowly never. Not soon will we see his like again.

Thus might I go on multiplying instances of how faithful our profession has been to the trust confided to it. I might speak to you of the lives of those great Scotch anatomists and physiologists, the Hunters and Bells, or of the even greater practical results which followed from the investigations of Richard Bright; from the discovery of Schoenlein that a disease of the skin known as favus was caused by parasitic fungus we might trace the great advances which have been made in our knowledge of the cause of disease in recent years, and see what vast good this knowledge is capable of accomplishing in the hands of a Lister or a Pasteur; or I might tell you of the discovery of that priceless blessing, anesthesia, to which America proudly lays claim; gladly would I recall to you the life and deeds of our own Sims, to whom the science of gynecology owes everything, or relate the histories of Flint, Gross, Mitchell, Vaughan, and last, but among the greatest, that model physician, DaCosta. But it cannot be: already is the time arrived when we should part. But before we separate let me exhort you who are students of medicine to study well for the sake of your own happiness, as well as for the good of your fellow-man, the lives of these great disciples of truth and humanity of whom I have just spoken. From them you will gain a broader view of medicine than you can obtain from the study of books alone—an inspiration which can come only from the chosen of God. Heed well then the lessons which their lives teach, and let them serve as models for your own. Marching under the broad folds of their spotless banners well may each of you realize what has been so beautifully expressed by Leigh Hunt in these lines:

> Abou Ben Adhem (may his tribe increase!)
> Awoke one night from a deep dream of peace,
> And saw, within the moonlight in his room,
> Making it rich and like a lily bloom,
> An Angel, writing in a book of gold:—
> Exceeding peace had made Ben Adhem bold,
> And to the Presence in the room he said,
> "What writest thou?" The Vision raised its head,
> And, with a look made of all sweet accord,
> Answered, "The names of those that love the Lord."
> "And is mine one?" said Abou. "Nay, not so,"
> Replied the Angel. Abou spoke more low,
> But cheerily still, and said, "I pray thee, then,
> Write me as one that loves his fellow-men."
> The Angel wrote, and vanished. The next night
> It came again with a great wakening light,
> And showed the names of those whom love of God had blest,
> And lo! Ben Adhem's name led all the rest.

More than this I could not wish you.

